# Psychometric validation of the Korean version of PROMIS 29 Profile V2.1 among patients with lower extremity problems

**DOI:** 10.1186/s13102-021-00374-1

**Published:** 2021-11-24

**Authors:** Youngha Kim, Danbee Kang, Eunjee Kang, Jihyun Lim, Sooyeon Kim, Heesu Nam, Sungkeun Shim, Mangyeong Lee, Young-Wan Moon, Seung-Jae Lim, Ki-Sun Sung, Juhee Cho

**Affiliations:** 1 Center for Clinical Epidemiology, Samsung Medical Center, Seoul, Republic of Korea; 2 Department of Clinical Research Design and Evaluation, SAIHST, Sungkyunkwan University, 81 Irwon-ro, Gangnam-Gu, 06351 Seoul, South Korea; 3 Department of Medical Device Management and Research, SAIHST, Sungkyunkwan University, Seoul, Republic of Korea; 4 Department of Digital Health, SAIHST, Sungkyunkwan University, Seoul, Republic of Korea; 5 Departments of Epidemiology and Health, Behavior and Society, Johns Hopkins Bloomberg School of Public Health, Baltimore, MD USA; 6 Department of Orthopedic Surgery, Samsung Medical Center, Sungkyunkwan University School of Medicine, 81 Irwon-ro, Gangnam-Gu, 06351 Seoul, South Korea

**Keywords:** Quality of life, Function, Lower extremity problems, validation, PROMIS-29

## Abstract

**Background:**

Patients with lower extremity problems (LEP) commonly experience functional loss, pain, decreased range of motion, inadequacy in daily living activities, and structural change in radiographic evaluations. However, the traditional patient-reported outcome measurement which focused on symptoms, had a limited scope of applicability. This study aimed to validate the psychometric properties of the Korean version of PROMIS-29 Profile v2.1 (K-PROMIS-29 V2.1), a multi-dimensional measure for assessing generic profile health-related quality-of-life (HRQoL) in a sample of patients with lower extremity problems (LEP).

**Methods:**

Participants were recruited from the orthopedic outpatient clinics at the Samsung Medical Center in Seoul, South Korea from September to October 2018. Participants completed a survey questionnaire that included the K-PROMIS-29 V2.1 and the SF-36v2. Principal component analysis (PCA) and confirmatory factor analysis (CFA) and Pearson’s correlations were used to evaluate the reliability and validity of the K-PROMIS-29 V2.1.

**Results:**

A total of 299 participants were enrolled in the study and 258 (86%) completed the study questionnaire. The mean age (SD) of the participants was 56.6 (14.5) and 32.3%, 29.8, and 25.2% of the study participants visited outpatient clinics for foot, knee, and hip problems respectively. The Cronbach’s alpha coefficients of 7 sub-domains in K-PROMIS-29 V2.1 ranged from 0.80 to 0.95, indicating satisfactory internal consistency. In CFA, the goodness-of-fit indices were high (CFI = 0.937 and SRMR = 0.061). High to moderate correlations were found between comparable subscales of the K-PROMIS-29 V2.1 and subscales of the SF-36v2 (r = 0.55–0.70).

**Conclusions:**

The K-PROMIS-29 V2.1 is a reliable and valid measure for assessing a broad range of health-related quality-of-life domains in patients with LEP. It would reflect the real-life symptoms experienced by patients with LEP.

**Supplementary Information:**

The online version contains supplementary material available at 10.1186/s13102-021-00374-1.

## Background

With an increasing prevalence of aging and obesity, the number of individuals affected by lower extremity problems (LEP), including osteoarthritis, sprain, strains, or tendinitis in hip, knee, ankle and foot, is expected to reach 50% of the population within the next 20 years [1, 2]. The outcomes of treatment for LEP can be assessed with various methods; implant survivorship, image-based assessment, clinical assessment and patient-reported outcome measures (PROMs). While the first three modalities are objective in nature, patient report can provide a subjective measure of the patients' perception of the success of an intervention [3]. In fact, the impact of severe LEP is multidimensional. LEP patients often experience functional loss, pain, decreased range of motion, inadequacy in daily living activities [4–6]. A thorough assessment of the utility of treatment for LEP should be more comprehensive than the approach we have applied in the past to other conditions, and therefore requires the application of a meaningful, appropriate PROMs [7, 8].

Many PROMs such as the Western Ontario and McMaster Universities Osteoarthritis (WOMAC) Index, or original Knee Injury and Osteoarthritis Outcomes Survey (KOOS) and physical tests are used to assess the outcome dimensions of the ICF among LEP patients. However, these traditional PROMs focused on pain and function, but that other domains like fatigue and psychosocial aspects are also important to measure, which were common problems among LEP patients [9–11]. Then, using multiple evaluation measures may be time consuming for patients, cost prohibitive, and impractical for busy clinical settings [12]. In addition, the purpose of LEP-specific measurements is to identify LEP related symptoms and functions that could make it difficult to compare with other diseases.

In fact, generic measures, such as the EuroQoL-5D (EQ-5D) [13] and the 36-item short-form health survey (SF-36) [14] were frequently used to assess the HRQoL in LEP patients [3]. However, EQ-5D had relatively larger ceiling effect than other HRQoL measures and do not discriminate well severe and mild LEP [3]. In 2010, National Institutes of Health (NIH) in the U.S. funded to develop the Patient-Reported Outcomes Measurement Information System (PROMIS) to unify measurement through standardized measures with broad applicability across health problems in clinical practice, research, and quality measurement. The PROMIS-29 is a multi-dimensional measure for assessing generic profile health-related quality-of-life (HRQoL) as the shortest version of PROMIS [15]. The PROMIS-29 covers frequently reported symptoms such as fatigue and sleep disturbance in LEP [16]. Compare to SF-36, the PROMIS-29 has better measurement properties than SF-36 [17]. The PROMIS-29 is available in 51 different languages [18] and it has been used to evaluate HRQoL in patient with chronic disease including arthritis, congestive heart failure, diabetes, osteoporosis, stroke, cancer, hemophilia and burn survivors [16, 19–22]. However, there was no psychometric validation study of the PROMIS-29 in patients with LEP. Thus, we aim to examine the validity of the PROMIS-29 among LEP patients.

## Methods

### Study participants and procedure

From September to October 2018, we conducted a cross-sectional survey at the orthopedics outpatient clinics at Samsung Medical Center in Seoul, South Korea. Patients were eligible for this study if they had lower extremity problems including osteoarthritis, sprain, strain, fracture, pain in hip, knee, foot and ankle, aged ≥ 18 years, and able to speak and read Korean. Trained researchers explained the purpose and procedures of the study to participants. Patients who reported any physical or psychiatric problem that would interfere with completing the questionnaire were excluded from the survey. After giving informed consent, participants were asked to complete the questionnaire on paper. This study was approved by the Institutional Review Board (IRB) of the Samsung Medical Center (IRB number: SMC-2017-03-103-012).

### Measurement

We used the Korean version of the PROMIS-29 Profile V2.1 (K-PROMIS-29 V2.1) and obtained the permission to use K-PROMIS-29 V2.1 from the Korean PROMIS National Center (PNC) of the PROMIS Health Organization [23]. The K-PROMIS-29 V2.1 was translated to Korean using the Functional Assessment of Chronic Illness Therapy (FACIT) Translation Methodology [24]. The PROMIS-29 V2.1 consists of 29 items in seven domains: physical function (four items), anxiety (four items), depression (four items), fatigue (four items), sleep disturbance (four items), ability to participate in social roles and activities (four items), pain interference (four items), and pain intensity (one item). We use a five-point Likert scale (range 1–5) to measure the severity or frequency of the symptoms. The single pain intensity item was scored separately and the response scale ranged from 0 (no pain) to 10 (worst pain imaginable). The questions regarding physical function and ability to participate in social roles and activities did not provide a specific time frame. For the other five domains, questions were asked about the past seven days. Then we converted the item score into t-scores which is standardized for the general US population [mean (SD) 50 (10)] using a T-score metric via Assessment Center [15, 25]. When we calculated the T-score, we included all participants, even those with missing values, using the algorithm from the Assessment Center [15]. Higher T-scores represent better physical function, ability to participate in social roles and activities. In terms of symptoms, higher score indicates more severe levels of anxiety, depression, fatigue, sleep disturbance, pain interference and pain intensity.

To examine convergent and discriminant validity, we used the Short Form Health Survey version-2.0 (SF-36v2), which is the most widely used method for measuring generic health status with a 4-week recall period. The SF-36v2 is comprised of 36 items in eight domains: physical functioning (10 items), role limitations due to physical functioning (four items), bodily pain (two items), general health perceptions (five items), vitality (four items), social functioning (two items), role limitations due to emotional functioning (three items), and mental health (five items). The physical functioning items form a hierarchical Guttman scale, in which each item consistently decreases in severity or difficulty. All items were rated on Likert-type or frequency response scales, ranging from three response categories for physical functioning items to six response categories for bodily pain items. Using the standard scoring algorithm, scales scores were linearly transformed to range from 0 to 100, with higher scores representing better health status [26]. The validity and reliability of the SF-36v2 has been well established in the Korean language [27–29].

We also asked study participants about their socio-demographic characteristics, including marital status, education level, monthly family income, and working status. Clinical characteristics were obtained from electronic medical records.

### Statistical analysis

The analysis was conducted using the T-scores. To assess the reliability of the K-PROMIS-29 V2.1, we calculated the internal consistency of each domain using Cronbach’s α and the item-rest correlation of each item. It is generally accepted that an α of 0.6–0.7 indicates an acceptable level and 0.8 or greater indicates a very good level of reliability [30].

To confirm the construct validity, a Principal Component Analysis (PCA) was performed to determine the underlying structure of the K-PROMIS-29 V2.1. After extracting factors that had an eigenvalue > 1 using scree plot, we performed a principle axis factor procedure with a varimax rotation to extract latent constructs to simplify the loadings of items by removing the middle ground and more specifically identifying the factor upon which data load. Furthermore, we carried out confirmatory factor analysis (CFA) using the maximum likelihood to test whether our factor structure fit the data. Several goodness-of-fit indices were used to evaluate the model fit, including comparative-fit-index (CFI), standardized root mean-squared residual (SRMR), and root mean square error of approximation (RMSEA). A CFI > 0.9, SRMR < 0.08, and RMSEA < 0.06 indicate a good fit to the data [31].

To examine convergent and discriminant validity, hypotheses on the direction and magnitude of Pearson’s correlations between the K-PROMIS-29 V2.1 and SF-36v2 were formulated a priori [32]. We expected moderate (0.5 <|r|< 0.7) or strong correlations (|r|≥ 0.7) between conceptually similar domains in the K-PROMIS-29 V2.1 and SF-36v2 as convergent validity (marked in grey in Table 4) [33]. We completed the pairwise deletion in the analysis.

All significance tests were two-tailed and *P* < 0.05 was considered significant. All data analyses were performed using STATA version 15 (StataCorp LLC, College station, USA).

## Results

### Study participants

A total of 299 participants were enrolled in the study and 258 (86%) completed the study questionnaire. Among the 41 patients who were excluded from the study due to missing PROMIS 29 items, 21 (51.2%), 7 (17.1%), 5 (12.2%), 8 (19.5%) had not answered 1, 2, 3 and more than 4 items, respectively. Participants did not answer the question about their sleep quality most frequently (4%).

Of the 258 participants, 153 (59.3%) were female with a mean age (SD) of 56.6 (14.5). Among the participants, 21.0% had completed less than a middle school education (Table 1). The type of diseases that participants had included hip (n = 65, 25.2%), knee (n = 77, 29.8%), ankle and foot (n = 91, 32.3%), and others (n = 25, 9.7%). Participants with knee problems were most likely to be female and older than most other participants (Additional file 1: Table 1). In the T-scores, the study sample reported lower physical functioning (mean 45.9, SD 9.0) and higher pain (mean 58.0, SD 8.5) than the general population [15].

**Table 1:** Characteristics of study population (N = 258)

Characteristics*	Patients
	(N = 258)
Sex (female)	153 (59.3)
Age (years)	56.6 (14.5)
Age categories	
< 50	72 (27.9)
50 to < 60	57 (22.1)
60 to < 70	87 (33.7)
≥ 70	42 (16.3)
Marital status	
Single	30 (11.7)
Married	196 (76.6)
Divorced/Bereavement	30 (11.7)
Living alone (yes)	27 (10.5)
Education level	
≤ Middle school	54 (21.0)
High school	78 (30.4)
≥ More than college	125 (48.6)
Monthly family income	
< $2000	62 (24.7)
$ 2000–$3990	59 (23.5)
≥ $4000	130 (51.8)
Current working status (yes)	139 (54.1)
Smoking status (current smoker)	24 (9.4)
Drinking status (current drinker)	98 (38.6)
Type of disease	
Hip	65 (25.2)
Knee	77 (29.8)
Ankle and foot	91 (32.3)
Others	25 (9.7)

*Values presented as n (%) or mean (SD). In this data set, education level (n = 1), current worker (n = 1), living alone (n = 1), marital status (n = 2), smoking status (n = 2), drinking status (n = 4), and monthly family income (n = 7) had missing data. For all other variables, the values were available for all participants

### Internal consistency reliability

The Cronbach’s alpha coefficients of 7 sub-domains in the K-PROMIS-29 V2.1 ranged from 0.80 to 0.95, indicating satisfactory internal consistency. The pain interference had the highest Cronbach’s α (0.95). Item-rest correlations, when any one of the items was removed, varied from 0.45 to 0.91. While all the items had generally accepted levels of item-rest correlation (≥ 0.60), the item “In the past 7 days, my sleep was refreshing” had a relatively low correlation (r = 0.50) with other items in sleep disturbance (Table 2).

**Table 2:** T-score and internal consistency reliability of PROMIS-29 Profile V2.1 domain

	T-score Mean (SD)	Cronbach's α	Item-rest correlation
**Physical function**	45.9 (9.0)	0.91	
Are you able to do chores such as vacuuming or yard work?			0.71
Are you able to go up and down stairs at a normal pace?			0.78
Are you able to go for a walk of at least 15 min?			0.83
Are you able to run errands and shop?			0.88
**Anxiety**	50.5 (9.1)	0.87	
In the past 7 days, I felt fearful			0.73
In the past 7 days, I found it hard to focus on anything other than my anxiety			0.70
In the past 7 days, my worries overwhelmed me			0.74
In the past 7 days, I felt uneasy			0.74
**Depression**	49.5 (7.7)	0.85	
In the past 7 days, I felt worthless			0.67
In the past 7 days, I felt helpless			0.73
In the past 7 days, I felt depressed			0.69
In the past 7 days, I felt hopeless			0.67
**Fatigue**	47.0 (8.8)	0.88	
During the past 7 days, I feel fatigued			0.71
During the past 7 days, I have trouble starting things because I am tired			0.67
In the past 7 days, how run-down did you feel on average?			0.77
In the past 7 days, how fatigued were you on average?			0.81
**Sleep Disturbance**	50.8 (8.5)	0.80	
In the past 7 days, my sleep quality was…			0.62
In the past 7 days, my sleep was refreshing			0.50
In the past 7 days, I had a problem with my sleep			0.69
In the past 7 days, I had difficulty falling asleep			0.64
**Ability to participate in social roles and activities**	51.2 (10.7)	0.94	
I have trouble doing all of my regular leisure activities with others			0.82
I have trouble doing all of the family activities that I want to do			0.84
I have trouble doing all of my usual work (include work at home)			0.84
I have trouble doing all of the activities with friends that I want to do			0.89
**Pain Interference**	58.0 (8.5)	0.95	
In the past 7 days, how much did pain interfere with your day to day activities?			0.84
In the past 7 days, how much did pain interfere with work around the home?			0.91
In the past 7 days, how much did pain interfere with your ability to participate in social activities?			0.89
In the past 7 days, how much did pain interfere with your household chores?			0.88
**Pain intensity**			

### Construct validity

In the PCA, the factor loadings for the six retained and varimax rotated factors were obtained (Table 3). The variance explained by the six-factor solution was 74.6%. While other domains confirmed our hypothesis regarding the original constructs of the K-PROMIS-29 V2.1, “depression and fatigue” were combined as one domain and sleep disturbance items were separated by “sleep quality” and “sleep was refreshing” among others.

**Table 3:** Exploratory factor analysis

**Original domain and items**	Factor loading					
	**F1**	**F2**	**F3**	**F4**	**F5**	**F6**
**Physical Function**						
Chores such as vacuuming or yard work?	**0.84**	0.01	− 0.11	0.23	0.08	− 0.01
Go up and down stairs at a normal pace?	**0.80**	0.12	− 0.02	0.22	0.00	− 0.06
Go for a walk of at least 15 min?	**0.87**	0.04	− 0.08	0.21	0.04	− 0.03
Are you able to run errands and shop?	**0.90**	0.06	− 0.04	0.21	0.03	− 0.01
**Anxiety**						
In the past 7 days, I felt fearful	0.14	0.25	0.25	**0.75**	− 0.01	− 0.05
In the past 7 days, I found it hard to focus on anything other than my anxiety	0.11	0.29	0.40	**0.62**	0.07	− 0.09
In the past 7 days, my worries overwhelmed me	0.07	0.15	0.31	**0.76**	0.01	0.01
In the past 7 days, I felt uneasy	0.12	0.21	0.45	**0.68**	0.09	− 0.04
**Depression**						
In the past 7 days, I felt worthless	0.08	0.00	**0.65**	0.39	0.00	− 0.08
In the past 7 days, I felt helpless	0.09	0.25	**0.71**	0.29	− 0.01	− 0.07
In the past 7 days, I felt depressed	0.05	0.18	**0.70**	0.36	0.02	− 0.08
In the past 7 days, I felt hopeless	0.03	0.06	**0.72**	0.23	0.06	− 0.01
**Fatigue**						
During the past 7 days, I feel fatigued	0.11	0.34	**0.64**	0.16	0.17	− 0.05
During the past 7 days, I have trouble starting things because I am tired	0.13	0.43	**0.53**	0.25	0.26	0.02
In the past 7 days, how run-down did you feel on average?	0.00	0.42	**0.60**	0.18	0.20	− 0.01
In the past 7 days, how fatigued were you on average?	0.02	0.38	**0.70**	0.06	0.25	− 0.03
**Sleep disturbance**						
In the past 7 days, my sleep quality was	− 0.15	− 0.07	− 0.04	− 0.03	− 0.16	**0.87**
In the past 7 days, my sleep was refreshing	− 0.02	− 0.08	− 0.06	− 0.04	− 0.04	**0.90**
In the past 7 days, I had a problem with my sleep	0.02	0.15	0.12	− 0.01	**0.92**	− 0.11
In the past 7 days, I had difficulty falling asleep	0.00	0.11	0.07	0.05	**0.95**	− 0.05
**Ability to participate in social roles and activities**						
I have trouble doing all of my regular leisure activities with others	**0.81**	0.19	0.17	− 0.14	− 0.02	− 0.05
I have trouble doing all of the family activities that I want to do	**0.83**	0.20	0.20	− 0.09	− 0.04	− 0.05
I have trouble doing all of my usual work (include work at home)	**0.88**	0.21	0.10	− 0.08	− 0.03	− 0.06
I have trouble doing all of the activities with friends that I want to do	**0.84**	0.26	0.19	− 0.13	0.01	− 0.03
**Pain interference**						
In the past 7 days, how much did pain interfere with your day to day activities?	0.18	**0.86**	0.08	0.18	0.11	− 0.05
In the past 7 days, how much did pain interfere with work around the home?	0.19	**0.89**	0.15	0.07	0.08	− 0.05
In the past 7 days, how much did pain interfere with your ability to participate in social activities?	0.18	**0.86**	0.26	0.11	0.10	− 0.01
In the past 7 days, how much did pain interfere with your household chores?	0.19	**0.86**	0.20	0.09	0.07	− 0.04
**Pain intensity**	0.11	**0.75**	0.05	0.15	0.08	− 0.14

The highest loading has been highlighted in bold for each item

**Fig. 1:**
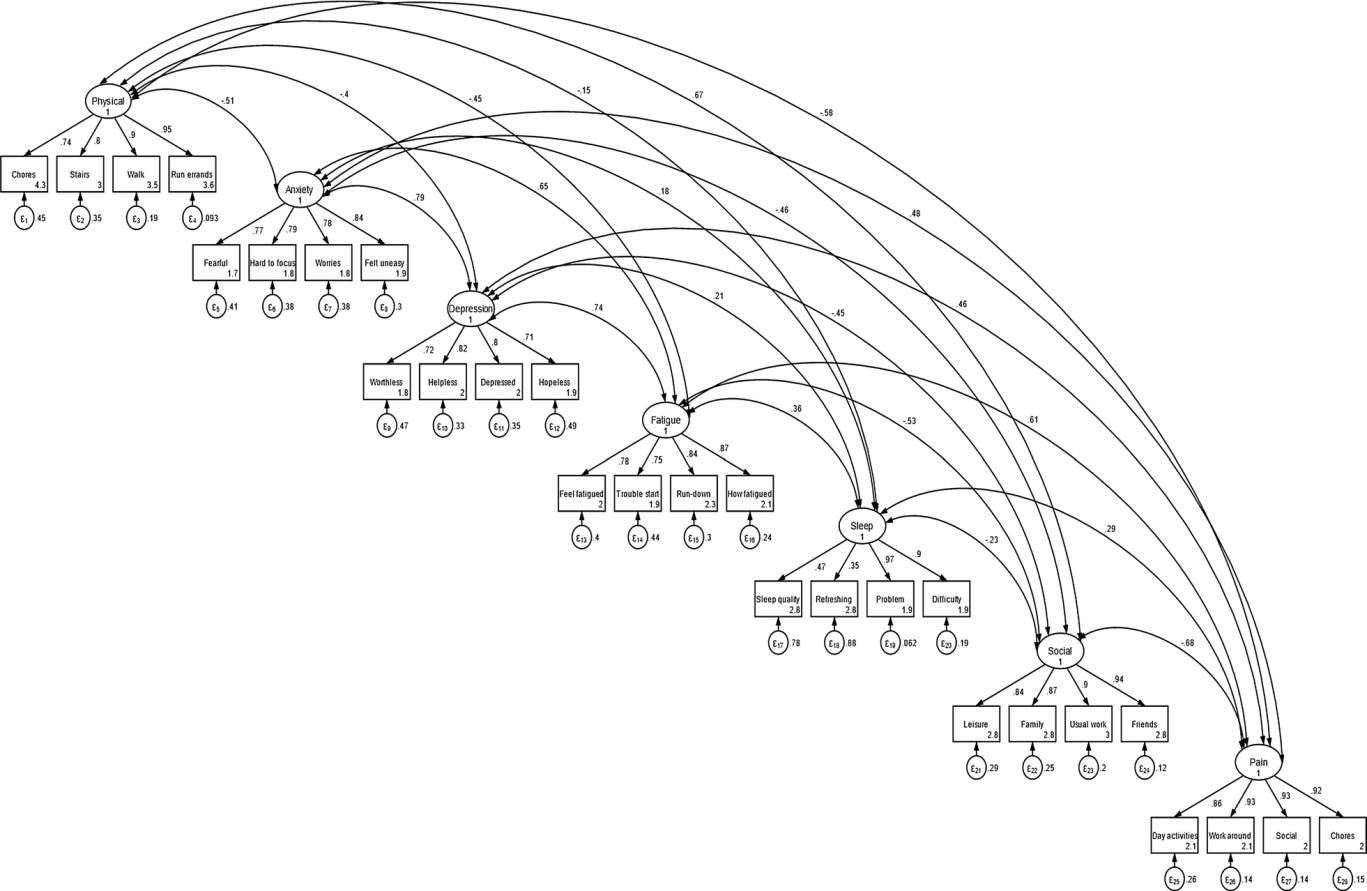
Confirmatory factor analysis of the PROMIS-29 V2.1 items

In confirmatory factor analysis, the goodness-of-fit indices for the K-PROMIS-29 V2.1 (Fig. 1) were high (CFI = 0.937, SRMR = 0.061, and RMSEA = 0.065). However, “sleep quality” and “sleep was refreshing” in the sleep domain had a relatively large rate of error. Regarding the correlation between the domain in K-PROMIS-29 V2.1, depression had a high correlation with anxiety and fatigue (0.79 and 0.73, respectively). However, the factorial correlation between sleep disturbance and the rest domains was relatively low.

### Convergent validity

In the convergent and discriminant validity of the K-PROMIS-29 V2.1, 9 domains out of 9 (100%) which expected more than moderate correlation had 0.5 or more correlated and 41 domains out of 47 which expected had lower correlation had less than 0.5 correlated. Pain interference in K-PROMIS-29 V2.1 and bodily pain in SF-36v2 observed a large correlation (*r* = − 0.70). In addition, the correlations between physical function in K-PROMIS-29 V2.1 and physical functioning (r = 0.58), and role-physical (r = 0.57) in SF-36v2 were moderate. Regarding the ability to participate in social roles and activities in K-PROMIS-29 V2.1, there were moderate correlations observed with role-physical (r = 0.60), social functioning (r = 0.55), and role-emotional (r = 0.54) in SF-36v2. Anxiety (r = − 0.58) and depression (r = − 0.62) in K-PROMIS-29 V2.1 were also moderately correlated with mental health in SF-36v2. As we expected, sleep disturbance was weakly correlated with SF-36v2 in all sub-domains. Nonetheless, general health in SF-36v2 was weakly correlated with K-PROMIS-29 V2.1 across all sub-domains (Table 4).

**Table 4:** Pearson’s correlation coefficients comparing the PROMIS-29 V2.1 with SF-36v2 (N = 258)

SF-36v2	K-PROMIS-29 V2.1						
	Physical Function	Ability to Participate in Social Roles and Activities	Anxiety	Depression	Fatigue	Pain Interference	Sleep Disturbance
Physical functioning	**0.58**	0.52	− 0.31	− 0.28	− 0.39	− 0.60	− 0.29
Role-physical	**0.57**	**0.60**	− 0.35	− 0.30	− 0.44	− 0.57	− 0.24
Role-emotional	0.47	**0.54**	− 0.42	− 0.44	− 0.50	− 0.52	− 0.27
Social functioning	0.51	**0.55**	− 0.38	− 0.41	− 0.44	− 0.55	− 0.24
Mental health	0.32	0.33	**− 0.58**	**− 0.62**	− 0.51	− 0.41	− 0.37
Vitality	0.35	0.42	− 0.42	− 0.45	**− 0.59**	− 0.49	− 0.35
Bodily pain	0.44	0.47	− 0.35	− 0.27	− 0.45	**− 0.70**	− 0.26
General health	0.23	0.31	− 0.31	− 0.33	− 0.45	− 0.43	− 0.38

In this data set, role-physical (n = 1) and role-emotional (n = 1) had missing data

The expected associated domain between similar domains in K-PROMIS-29 V2.1 and SF-36v2 was highlighted in bold

## Discussion

In this study, the Korean version of PROMIS-29 V2.1 was found to be a reliable and valid measure of quality of life among patients with LEP. The goodness-of-fit indices for the original domain of K-PROMIS-29 V2.1 were also high. The convergent validity of the PROMIS-29 V2.1 was demonstrated by its varying degree of correlations with SF-36v2.

In total, 85% of participants completed all the questions, which is a higher completion rate than that reported in other studies [34, 35]. Considering that more than 16% of the study participants were over 70 years and 20% had less than a middle school education, the K-PROMIS-29 V2.1 seems to be a feasible instrument in evaluating HRQoL, regardless of age and literacy. In our study, the most commonly unanswered question by study participants was about sleep quality (n = 12, 4%). Participants might have missed this item because the question was formatted differently from others. The other questions were complete statements or questions that participants responded to using a Likert scale (e.g. I feel fatigued. Response options: Not at all, A little bit…Very much). However, the sleep quality question was an open-ended question written as, “My sleep quality was…” and participant were asked to choose the response that best described their sleep quality (Very poor, Poor, Fair, Good, Very good). In fact, in a previous study conducted in Dutch [36], over 90% of the study participants marked “My sleep quality was…” as being one of the most difficult items to answer. The authors hypothesized that the item might be difficulty to understand because of the other response options [36].

Results indicated that the internal consistency reliability of the measure is high. Cronbach’s α for all subdomains fell in the range of acceptable internal consistency [37].

The confirmatory factor analysis also confirmed our hypothesis regarding the original constructs of the K-PROMIS-29 V2.1 except in the sleep disturbance subdomain. In this study, the item regarding sleep quality had relatively low item-rest correlations with the other items: “In the past 7 days, I had a problem with my sleep” and “In the past 7 days, I had difficulty falling asleep.” These items also had a large margin of error in the confirmatory factor analysis. In fact, they were related to a different factor in exploratory factor analysis. In a previous study, sleep initiation and sleep continuity appeared as separate constructs, and people perceived feeling refreshed in the morning and good sleep continuity as good sleep [38]. Similarly, our study participants perceived or interpreted questions about “a problem with sleep” and “difficulty with falling asleep” as questions about “sleep initiation” and questions about “sleep quality” and “refreshment of sleep” as questions about “sleep quality,” which is strongly related to sleep continuity [38].

The convergent validity of the K-PROMIS-29 V2.1 was demonstrated by its varying degree of correlation with the SF-36v2. The K-PROMIS-29 V2.1 domain correlated with the comparable SF-36v2 subdomain. In addition, as we expected, sleep for which no comparable SF-36v2 element had low correlated with SF-36v2 in all sub-domains. In patients with lower extremity problems, sleep disturbance is considered a core aspect of HRQoL. Previous studies suggest that at least half of patients with lower extremity problems report significant sleep disturbance [9, 39], with some studies indicating the prevalence may be as high as 70% [9, 39]. Sleep problems negatively impact pain, psychological health, and quality of life [40, 41]. However, only a limited number of HRQoL studies have assessed the sleep problems of patients with lower extremity problems because most previous studies have used general quality of life measures which did not include the sleep domain as done in SF-36 [26]. Therefore, for the future studies, it is recommended to use the K-PROMIS-29 V2.1 for evaluating quality of life of patients with LEP.

There are several limitations to this study. First, we recruited only individuals who were visiting an orthopedic clinic at one institution in Korea, hence these findings may not be generalizable to patients in other settings. However, this study included patients with hip (25.2%), knee (29.8%), ankle and foot (32.3%) problems, and thus covered the entire area of the lower extremity. In addition, to test validity in participants who had low literacy level, we included approximately 20% of participants with very little education by the guideline from the FACIT Methodology [24]. Considering characteristics of our study participants, the K-PROMIS-29 V2.1 has acceptable measurement properties for use in patients from diverse backgrounds with lower extremity problems. Second, the study did not include an existing questionnaire that effectively measured sleep disturbance to confirm the convergent validity of the sleep disturbance sub-domain in the PROMIS-29 V2.1. However, previous studies have supported [42] the idea that sleep disturbance in PROMIS-29 V2.1 is highly correlated with Pittsburgh Sleep Quality Index [43].

## Conclusions

This study adds to the evidence base supporting the reliability and validity of K-PROMIS-29 V2.1 in assessing quality of life among Korean speakers being treated for lower extremity problems. The PROMIS-29 V2.1 is meant to be an efficient means of assessing a broad range of HRQoL domains, which evaluate physical function, anxiety, depression, fatigue, ability to participate in social roles and activities, sleep disturbance, and pain [44]. Although K-PROMIS-29 V2.1 included a comprehensive domain to measure the patients’ health status, since PROMIS measures are not condition-specific, researchers and clinicians have been reluctant to incorporate them in place of common legacy measures [39]. In addition, clinicians have hesitated to use PROMIS because the minimally clinically important difference for patients with lower extremity problems has yet to be established [40]. Therefore, studies with a larger and more representative sample using the K-PROMIS-29 V2.1 are necessary to assess the HRQoL of people with lower extremity problems more comprehensively. In addition, future studies should also test the responsiveness of PROMIS measures, and especially compare the responsiveness of PROMIS to the responsiveness of disease-specific PROMs.

## Supplementary Information

**Additional file 1: Table 1.** Characteristics of study participants by type of disease.

## Data Availability

The datasets generated and/or analyzed during the current study are not publicly available due to limitations of ethical approval involving the patient data and anonymity but are available from the corresponding author on reasonable request.

## Abbreviations

LEP: Lower extremity problems
PRO: Patient reported outcome
ICF: International classification of functioning, disability and health
PROM: Patient reported outcome measures
WOMAC: Western Ontario and McMaster Universities Osteoarthritis
KOOS: Knee injury and Osteoarthritis Outcomes Survey
PROMIS-29: Patient-Reported Outcome Measurement Information System 29
HRQOL: Health-related quality-of-life
NIH: National Institutes of Health
IRB: Institutional Review Board
K-PROMIS-29 V2.1: Korean version of the PROMIS-29 Profile V2.1
FACIT: Functional Assessment of Chronic Illness Therapy
SD: Standard deviation
SF-36v2: Short Form Health Survey version-2.0
CFA: Confirmatory factor analysis
GFI: Goodness-of-fit index
CFI: Comparative-fit-index
SRMR: Standardized root mean-squared residual
