# Supplementary material for: Psychometric validation of the Korean version of PROMIS 29 Profile V2.1 among patients with lower extremity problems
Source: BMC Sports Sci Med Rehabil. 2021 Nov 24;13:148. doi: 10.1186/s13102-021-00374-1 (PMC8614031; doi:10.1186/s13102-021-00374-1)
Supplement: Supplementary file 1 — Additional file 1: Table 1. Characteristics of study participants by type of disease. [file 13102_2021_374_MOESM1_ESM.docx]

**Supplementary Table 1. Characteristics of study participants by type of disease**

| **Characteristics** | **Hip** | **Knee** | **Ankle and foot** | **Other** | **P-value** |
| --- | --- | --- | --- | --- | --- |
|  | **(N = 65)** | **(N = 77)** | **(N = 91)** | **(N = 25)** |  |
| **Sex (female)** | 36 (55.4) | 64 (83.1) | 43 (47.3) | 10 (40.0) | <0.01 |
| **Age (years)** | 52.7 (15.3) | 65.1 (10.4) | 53.6 (14.1) | 51.9 (14.0) | <0.01 |
| **Age categories** |  |  |  |  | <0.01 |
| <50 | 27 (41.5) | 5 (6.5) | 30 (33.0) | 10 (40.0) |  |
| 50–<60 | 12 (18.5) | 11 (14.3) | 27 (29.7) | 7 (28.0) |  |
| 60–<70 | 18 (27.7) | 38 (49.4) | 24 (26.4) | 7 (28.0) |  |
| ≥70 | 8 (12.3) | 23 (29.9) | 10 (11.0) | 1 (4.0) |  |
| **Marital status** |  |  |  |  | <0.01 |
| Single | 14 (22.2) | 2 (2.6) | 11 (12.1) | 3 (12.0) |  |
| Married | 40 (63.5) | 61 (79.2) | 76 (83.5) | 19 (76.0) |  |
| Divorced/Bereavement | 9 (14.3) | 14 (18.2) | 4 (4.4) | 3 (12.0) |  |
| **Living alone (yes)** | 8 (12.5) | 7 (9.1) | 8 (8.8) | 4 (16.0) | 0.68 |
| **Education level** |  |  |  |  | 0.06 |
| ≤ Middle school | 8 (12.5) | 22 (28.6) | 20 (22.0) | 4 (16.0) |  |
| High school | 25 (39.1) | 25 (32.5) | 19 (20.9) | 9 (36.0) |  |
| ≥ More than college | 31 (48.4) | 30 (39.0) | 52 (57.1) | 12 (48.0) |  |
| **Monthly family income** |  |  |  |  | 0.54 |
| < $2,000 | 13 (20.3) | 24 (32.4) | 20 (22.7) | 5 (20.0) |  |
| $2,000–$3,990 | 17 (26.6) | 17 (23.0) | 21 (23.9) | 4 (16.0) |  |
| ≥ $4,000 | 34 (53.1) | 33 (44.6) | 47 (53.4) | 16 (64.0) |  |
| **Current work (yes)** | 40 (62.5) | 35 (45.5) | 49 (53.8) | 15 (60.0) | 0.21 |
| **Current smoker (yes)** | 11 (17.2) | 4 (5.2) | 6 (6.7) | 3 (12.0) | 0.07 |
| **Current drinker (yes)** | 24 (37.5) | 24 (31.6) | 38 (42.2) | 12 (50.0) | 0.33 |

*Values presented as n (%) or mean (SD). In this data set, education level (n =1), current worker (n =1), living alone (n = 1), marital status (n = 2), smoking status (n = 2), drinking status (n = 4), and monthly family income (n = 7) had missing data. For all other variables, the values were available for all participants.
